# The Meaningfulness of Effect Sizes in Psychological Research: Differences Between Sub-Disciplines and the Impact of Potential Biases

**DOI:** 10.3389/fpsyg.2019.00813

**Published:** 2019-04-11

**Authors:** Thomas Schäfer, Marcus A. Schwarz

**Affiliations:** Department of Psychology, Chemnitz University of Technology, Chemnitz, Germany

**Keywords:** effect size, Cohen, statistical power, sample size, replicability

## Abstract

Effect sizes are the currency of psychological research. They quantify the results of a study to answer the research question and are used to calculate statistical power. The interpretation of effect sizes—when is an effect small, medium, or large?—has been guided by the recommendations Jacob Cohen gave in his pioneering writings starting in 1962: Either compare an effect with the effects found in past research or use certain conventional benchmarks. The present analysis shows that neither of these recommendations is currently applicable. From past publications without pre-registration, 900 effects were randomly drawn and compared with 93 effects from publications with pre-registration, revealing a large difference: Effects from the former (median *r* = 0.36) were much larger than effects from the latter (median *r* = 0.16). That is, certain biases, such as publication bias or questionable research practices, have caused a dramatic inflation in published effects, making it difficult to compare an actual effect with the real population effects (as these are unknown). In addition, there were very large differences in the mean effects between psychological sub-disciplines and between different study designs, making it impossible to apply any global benchmarks. Many more pre-registered studies are needed in the future to derive a reliable picture of real population effects.

## Introduction

Research in psychology, as in most other social and natural sciences, is concerned with effects. Typically, effects relate to the variance in a certain variable across different populations (is there a difference?) or to the strength of covariation between different variables in the same population (how strong is the association between *x* and *y*?). Although there are other classes of typical parameters (e.g., means or proportions), psychologists have focused on differences and covariances in most of their investigations. As effects are the most frequent inducement of psychological research, scientific articles, textbooks, and conference presentations should be informative about their magnitude after empirical data have been collected. Thus, typically, an *effect size*—an often standardized measure of the magnitude of a certain phenomenon of scientific interest—is reported.

The effect size measures developed in recent decades (see, e.g., Kirk, 1996, 2007; Ellis, 2010; Fritz et al., 2012) have been used to provide a direct answer to the research questions that motivate a study (see also Pek and Flora, 2017, for a tutorial on how to report effect sizes in original psychological research). An effect size can be defined as “*a quantitative reflection of a magnitude of some phenomenon that is used for the purpose of addressing a question of interest*” (Kelley and Preacher, 2012, p. 140; emphasis in original) or, more simply, “an *effect size* (ES) is the amount of anything that’s of research interest” (Cumming and Calin-Jageman, 2017, p. 111; emphasis in original). Whereas the reporting of effect sizes in psychological papers was originally only a neat extra, it has become standard to provide effect sizes—together with a measure of their precision, such as confidence intervals—solely or as a supplement to inferential statistics (particularly, significance tests). In fact, the reporting of effect sizes and confidence intervals has been explicitly required by American Psychological Association [APA] (2001, 2010) publications for many years (see Appelbaum et al., 2018, for the latest Journal Article Reporting Standards for Quantitative Research in Psychology). While compliance in four prominent APA journals in 1995 was only 48% (Kirk, 1996), it was almost 100% in 2015 (Schäfer, 2018), for articles reporting an inferential statistic.

The most important advantage of most types of effect sizes is their independence from sample size so that they can express the size of an effect regardless of the size of the study. They also avoid the difficult—and often arbitrary—logic of inferential statistics (in particular, significance testing) but are more tied to the magnitude of what has been measured in a study and is used to estimate a specific population parameter. This is why effect sizes are not only the most useful way to answer a research question but are also used to calculate the statistical power of significance tests, for which the population parameter has to be determined before conducting a study. These two fields of application have always been concerned with the question of when an effect is small, medium, or large, or—to put it more simply—when an effect is meaningful or not. There have been two principal approaches to answering this very question. One is to compare the effect found in a study with the effects that have been found in previous studies in the respective area of research. Another is to apply global conventional benchmarks for small, medium, and large effects. In the present article, we show that both approaches are problematic. We also discuss under what conditions they might be applicable and what is needed in psychological research to set up these conditions. Before doing so, we briefly introduce why effect sizes are important for psychological research and how the question of the meaningfulness of effects has been traditionally answered.

### Using Effects Sizes to Answer the Research Question

Practically every empirical study is looking for an effect. Effect sizes quantify the magnitude of the effect that emerges from the sampled data. Thus, they are the currency of psychological research. Effect sizes can be unstandardized measures such as the difference between two means, but more often they are standardized, which makes them independent of a study’s scales and instruments, making it in principal possible to compare different domains and approaches. This is especially relevant to the integration of psychological evidence in meta-analyses, which typically use effect sizes from comparable studies to arrive at more reliable estimates of population parameters. Recent discussions have emphasized the need for replication studies and the integration of their results to produce more conclusive evidence in both basic and applied psychological fields (e.g., Ottenbacher, 1996; Brandt et al., 2014; Cumming, 2014). This increases the value of calculating, reporting, and discussing effect sizes. Since standardized effect sizes typically are unit-less their interpretation ultimately leads to the question of what is a small, medium, or large effect.

### Using Effect Sizes to Calculate Statistical Power

When using significance testing to make conclusions about the generalizability of sample results, empirical studies must consider statistical power. To calculate power—or rather the sample size necessary to reach a certain level of power—one needs to set the size of the effect that is likely to be true for the population. One way to do this is to look at previous studies in the area of the current research and—given that a large number of studies can be found—derive a *mean* or *typical* effect (note that this is not recommended when only few studies can be found, because of the sampling error, or if publication bias is likely, for instance, when the true population effect is small). If this is not possible, one can rely on a conventional definition of small, medium, and large effects and pick one for the current power analysis. The latter approach is more convenient and thus also the most prominent (Sedlmeier and Gigerenzer, 1989). The requirement to estimate a population effect when conducting a power analysis again leads to the question of what is a small, medium, or large effect.

### When Is an Effect Small or Large? Cohen’s Approaches

When using effect sizes to quantify and share scientific insights and make sensible power calculations for reliable studies one is inevitably faced with the difficulty of saying when an effect is small or large. In a series of seminal contributions, Cohen (1962, 1969, 1977, 1988, 1990, 1992) developed the concept of power analysis in the behavioral sciences and thought deeply about conventional standards for the interpretation of effect sizes. Cohen (1988, p. 25) was aware that terms such as “‘small,’ ‘medium,’ and ‘large’ are relative, not only to each other, but to the area of behavioral science or even more particularly to the specific content and research method.” Consequently, he recommended deriving the judgment about small, medium, and large effects from the results of previous studies in the respective area of research. As a researcher would have to compare her current result with what has been found in previous studies we call this the *comparison* approach. However, Cohen was also aware that for most researchers it was more convenient to have a shortcut, that is, a broad conventional definition that could be used as a point of reference. Let us call this the *conventions* approach.

In order to derive specific conventions, Cohen (1988, p. 25) referred to real-world examples such as the body height of women and men and argued that a medium effect should “represent an effect likely to be visible to the naked eye of a careful observer,” which he saw in a value of *d* = 0.5, corresponding to *r* = 0.3 and η^2^ = 0.06. He set “small ES to be noticeably smaller than medium but not so small as to be trivial,” which he saw at *d* = 0.2, corresponding to *r* = 0.1 and η^2^ = 0.01. And he set “large ES to be the same distance above medium as small was below it,” yielding *d* = 0.8, corresponding to *r* = 0.5 and η^2^ = 0.14. Cohen was aware that any global conventions are problematic and (Cohen, 1962, p. 146) conceded “these values are necessarily somewhat arbitrary, but were chosen so as to seem reasonable. The reader can render his own judgment as to their reasonableness…, but whatever his judgment, he may at least be willing to accept them as conventional.”

### The Applicability of Cohen’s Approaches

Generations of psychologists have been adopting both the comparison and the conventions approach to interpret the effects of their own investigations and to conduct calculations of statistical power. Yet, both approaches are only useful and applicable under certain conditions. Specifically, the expediency of the comparison approach highly depends on the reliability of the information a researcher can get about the effects that have been ‘typically’ found in the respective area of research so far. Cohen’s highly sensible idea to refer to those past effects only works when those effects are more or less representative of the ‘true’ effects in the population. In other words, the effects that are available for a comparison must not be biased in any way in order to warrant a meaningful integration of a study result into a broader context of past research. By way of example, the effect of a newly developed psychological intervention against depression can only be meaningfully compared with effects from other interventions when those effects represent the true efficacy in the population.

The expediency of the conventions approach, on the other hand, where global conventional benchmarks might be suggested to represent small, medium, and large effects, depends on the homogeneity of different areas of psychological research. That is, the distribution of effects should be similar enough across different sub-disciplines in order to warrant the application of global conventions. Cohen based his judgments on examples from biology and developmental psychology but never undertook a systematic review of empirical effects—neither in this domain nor in others. He noted that his approach was “no more reliable a basis than [his] own intuition” (Cohen, 1988, p. 532). It should be mentioned again, however, that Cohen did not advocate the use of global conventions but saw these as a useful workaround when more detailed information is missing.

Can either of the two conditions—unbiased effects from past research and comparability of psychological sub-disciplines—be met by the existing empirical evidence? In the following, we suggest that they probably cannot. Specifically, effects are likely biased by the way empirical data are analyzed, reported, and published, and sub-disciplines are likely incommensurable in terms of the effects they typically reveal.

### The Impact of Analysis, Reporting, and Publication Biases on Effect Sizes in Psychology

In a perfect world, researchers would study an effect of interest with sound methods and publish and discuss their results regardless of their magnitude. In this ideal case, we could expect the distribution of all published effects to be a representative portrayal of what is there in the population. We would then also be able to compare the results of our own studies with the effects found in previous studies, at least within the realm of our respective areas of research. However, this ideal has become unattainable, at least since the so-called reproducibility crisis in psychology (Open Science Collaboration, 2012, 2015) and other disciplines such as medicine (Ioannidis, 2005). It was shown that many effects did not show up again in a replication (Open Science Collaboration, 2015). With regard to the effect sizes, the 95% confidence intervals of the replication effects contained the original effect in only 47.4% of the studies. More specifically, the mean effect decreased from *r* = 0.40 in the original studies to *r* = 0.20 in the replication studies. Similarly, in a more recent replication study, the median effect of 28 studies decreased from *d* = 0.60 in the original studies to *d* = 0.15 in the replication studies (Klein et al., 2018). The most important reasons discussed are questionable research practices (such as *p*-hacking, HARKing, intermediate testing, selective reporting of results) and the publication bias (small and non-significant effects are either not submitted for publication or are denied publication by reviewers or editors) (e.g., Bakker et al., 2012; John et al., 2012). These practices have very likely led to an inflation of the effects published in the psychological literature. Most impressively, this inflation of published effects often shows up in the course of meta-analyses where effects from very similar studies are combined, often revealing the absence of small, non-significant effects. Researchers have developed procedures such as trim-and-fill (Duval and Tweedie, 2000), *p*-curve (Simonsohn et al., 2014), and *p*-uniform (van Assen et al., 2015), some of which are quite efficient in uncovering bias in published effects, but none of which has proven sufficiently efficacious in quantifying and correcting for that bias (Renkewitz and Keiner, 2018). In other words, effects that have not been published are hard to reconstruct.

Yet, how large is the problem of inflated effects? As just mentioned, the Open Science Collaboration (2015) found that replication effects were half the magnitude of original effects. This gives enough reason not to rely on published effects when interpreting the effect of one’s own study. But the Open Science Collaboration’s focus on replication studies and use of only studies from high-ranked journals means there might not be sufficient information to reliably estimate the difference between published (i.e., potentially biased) effects and ‘true’ effects (i.e., effects representative of the population). In the present study, we employed a broader basis of empirical studies and compared the results of original research that has either been published traditionally (and might therefore be affected by the causes of bias just mentioned) or been made available in the course of a pre-registration procedure (therefore probably not affected by these biases).

### Differences Between Psychological Sub-Disciplines

When trying to compare Cohen’s conventions with published empirical effects, some researchers have collected effect sizes within several sub-disciplines. Some reviews found effect sizes to be larger than suggested by Cohen: Cooper and Findley (1982) found a mean *d* = 1.19 and a mean *r* = 0.48 from studies reported in social psychology textbooks. Haase et al. (1982) reported a median η^2^ = 0.08 from 701 articles in *Journal of Counseling Psychology*. Morris and Fritz (2013) reported a median η^2^ = 0.18 from 224 articles in memory research. Rubio-Aparicio et al. (2018) analyzed 54 meta-analyses/1,285 studies investigating the effectiveness of treatments in the field of clinical psychology and found a median *d* = 0.75 for standardized mean changes (i.e., within-subjects studies).

Other reviews found published effects to be smaller: Hemphill (2003) reported a middle *r* = 0.20–0.30 from 380 meta-analyses of treatment and assessment. Richard et al. (2003) reported a mean *r* = 0.21 from 322 meta-analyses/25,000 articles in social psychology. Gignac and Szodorai (2016) reported a median *r* = 0.19 from 87 meta-analyses/780 articles on individual differences. For standardized mean differences (i.e., between-subjects studies), Rubio-Aparicio et al. (2018; see above) found a median *d* = 0.41.

Some of these studies might have been selective in that they were covering only studies from textbooks that might be biased toward larger effects or referring only to one specific kind of effect size. But as a whole, they indicate that sub-disciplines might not be comparable. With our study, we made this question more explicit and collected representative data for the whole range of psychological sub-disciplines.

### The Present Study

In sum, our aim was (1) to quantify the impact of potential biases (e.g., analysis, reporting, and publication bias) on the magnitude of effect sizes in psychology as a whole and (2) to systematically investigate differences in the magnitude of effect sizes between psychological sub-disciplines. Aim 1 pertains to the comparison approach: If published effects are not representative of the effects in the population (as suggested by recent replication projects) it is problematic to infer the meaningfulness of an effect by looking at those published effects. Aim 2 pertains to the conventions approach: If the distributions of empirical effects differ strongly between sub-disciplines (see section “Differences Between Psychological Sub-Disciplines”) the use of any global conventions should be avoided. What is new to our approach is that (1) it is not limited to single studies/effects in specific areas (as in direct replication projects) but tries to employ a representative sample of psychological science as a whole and (2) it provides a direct and systematic comparison of different psychological sub-disciplines.

## Materials and Methods

There were three key methodological elements in our study. First, to get a representative overview of published effects in psychology, we analyzed a *random* selection of published empirical studies. Randomness ensured that each study had the same probability of being drawn, which is the most reliable path to generalizable conclusions. Second, to estimate how strongly published effects might be biased, we distinguished between studies with and without pre-registration. Third, to compare different sub-disciplines, we categorized the manifold branches of psychology into nine clusters and randomly drew and analyzed effects within each cluster. We now explain the procedure in more detail.

### Psychological Sub-Disciplines

To cover the whole range of psychological sub-disciplines we used the Social Sciences Citation Index (SSCI) that lists 10 categories for psychology: applied, biological, clinical, developmental, educational, experimental, mathematical, multidisciplinary, psychoanalysis, social. Our initial goal was to sample 100 effect sizes from each of these 10 categories, for 1,000 effect sizes in total. In the mathematical category, however, published articles almost exclusively referred to advances in research methods, not to empirical studies. It was not possible to sample 100 effect sizes, so this category was eventually excluded. Therefore, our selection of empirical effect sizes was based on the nine remaining categories, with a goal of 900 effect sizes.

### Representative Selection of Published Empirical Effects Without Pre-registration

For each category, the SSCI also lists relevant journals (ranging from 14 journals for psychoanalysis to 129 for multidisciplinary). Our random-drawing approach (based on the AS 183 pseudorandom number generator implemented in Microsoft Excel) comprised the following steps. (1) For each category, 10 journals were randomly drawn from those lists. (2) For each of these 90 journals, all volumes and issues were captured, from which 10 articles were then randomly drawn. (3) These 900 articles were read and analyzed as to their suitability for providing a measure of effect size for original research. We excluded theoretical articles, reviews, meta-analyses, methodological articles, animal studies, and articles without enough information to calculate an effect size (including studies providing non-parametric statistics for differences in central tendency and studies reporting multilevel or structural equation modeling without providing specific effect sizes). If an article had to be skipped, the random procedure was continued within this journal until 10 suitable articles were identified. If for a journal fewer than four of the first 10 draws were suitable, the journal was skipped and another journal within the category was randomly drawn. We ended up with 900 empirical effects representative of psychological research since its beginning (see Table 1). In this sample, there were no articles adhering to a pre-registration procedure. Sampling was conducted from mid 2017 till end of 2018. The data files with the statistical information extracted or calculated from the empirical articles, together with a documentation, can be accessed at https://osf.io/t8uvc/.

**Table 1:** Type, population, and design of the studies from which effects were obtained.

Descriptor	Studies without pre-registration		Studies with pre-registration	
	***N***	**%**	***N***	**%**
Type of study				
Experimental	177	20	44	47
Quasi-experimental	192	21	21	23
Other (e.g., correlational)	531	59	28	30
Population				
Clinical	204	23	1	1
Non-clinical	694	77	92	99
Mixed	2	0.2	0	0
Study design				
Between-subjects	388	43	66	71
Within-subject	498	55	27	29
Mixed	14	1.6	0	0

### Published Empirical Effects With Pre-registration

One of the most efficient methods to reduce or prevent publication bias and questionable research practices is pre-registration (e.g., Wagenmakers et al., 2012). More particularly, in the course of pre-registration, the theoretical foundation of an empirical study and its methodology together with a planned analysis protocol is registered and “frozen” at a public repository (such as Open Science Framework) before data collection. This procedure is suggested to avoid questionable research practices such as HARKing, *p*-hacking, or selectively analyzing. A particular form of pre-registration is *registered reports*, where the manuscript of an article excluding the “Results” and “Discussion” sections is submitted to a journal before data collection. If the manuscript is accepted, it is published regardless of the size and significance of the effect(s) it reports (so-called in-principle acceptance). Registered reports are the most effective way to also avoid publication bias; their effects can thus be considered to give a representative picture of the real distribution of population effects.

Since pre-registered studies have gained in popularity only in recent years, we did not expect there to be that many published articles adhering to a pre-registration protocol. We therefore set out to collect all of them instead of only drawing a sample. We used PsycINFO to search for the keywords ‘preregistered,’ ‘pre-registered,’ ‘preregistration,’ ‘pre-registration,’ and ‘registered report.’ Filtering out non-relevant articles left us with 93 original empirical articles in total, from which we could extract an effect size (see Table 1). Collection of studies was conducted from mid till end of 2018.

### Identifying the Key Research Question

We used the title and abstract of an article to identify the key research question. The first reported effect that unambiguously referred to that key research question was then recorded for that article. This was done to avoid including effects that simply referred to manipulation checks or any kind of pre-analysis, such as checking for gender differences. This protocol ensured that we ended up with 900 + 93 empirical effect sizes referring to 900 + 93 key research questions from the whole range of psychological research over the last about 100 years.

### Extracting and Transforming Effect Sizes

For most of the effects, a measure of effect size was provided directly in the article (56% for studies without pre-registration, 100% for studies with pre-registration). For the remaining effects, the effect size had to be calculated from the significance test statistics.

The most frequently reported effect sizes were Pearson’s *r*, Cohen’s *d*, and $\eta_{p}^{2}$. Because our aim was to get an impression of the distribution of effects from psychological science in general, we transformed all effect sizes to a common metric if possible. As the correlation coefficient *r* was the most frequently reported effect size and is often used as a common metric (e.g., in meta-analyses), we transformed effects to *r* whenever possible. Specifically, we transformed given effect sizes *d*, *g*, and partial eta-squared ($\eta_{p}^{2}$) to *r* and calculated *r* from *t* and *F* statistics for single effects when no effect size was given (see, e.g., Keppel, 1991; Lakens, 2013), resulting in 684 values for *r* in total for studies without pre-registration and 89 values for *r* in total for studies with pre-registration. Other effect sizes were less frequent and are not analyzed here: *R*^2^, *R*^2^_adjusted_, *w*, and odds ratio.

Because of the difference in how the error variance is calculated in between-subjects versus within-subject study designs it is actually not advisable to lump effects from one with effects from the other. This is particularly true for values of $\eta_{p}^{2}$ when these come from designs that might, for instance, additionally include covariates (Olejnik and Algina, 2003). However, this is often done when applying benchmarks for small, medium, and large effects. We therefore provide analyses for both the whole set of effects and the effects from between-subjects designs and within-subject designs separately.

### Comparing the Distributions of Published Effects With Cohen’s Benchmarks

The studies discussed in the Introduction (see section “Differences Between Psychological Sub-Disciplines”) took different approaches to comparing the distributions of effect sizes they had analyzed with Cohen’s conventional definitions of small, medium, and large effects. Some used the means and standard deviations of the distributions; others used the median and certain quantiles. We deemed it most sensible to divide the distributions of effect sizes into three even parts and take the medians of these parts (i.e., the 16.65%, 50%, and 83.35% quantiles) as the anchor points for small, medium, and large effects.

## Results

### Description of Studies

Effects came from articles published between 1912 and 2018, of course with many more being from recent years. The sample size ranged from 3 to 40,174 (*M* = 365, *SD* = 1,730, *Mdn* = 89, interquartile range = 164) for studies without pre-registration and from 20 to 120,115 (*M* = 470, *SD* = 667 [excluding the extreme value of 120,115], *Mdn* = 267, interquartile range = 425) for studies with pre-registration. See Table 1 for other descriptors and Table 2 for detailed statistics of the sample sizes separately for between-subjects designs and within-subject designs as well as sub-disciplines. With regard to between-subjects designs, the median and mean samples sizes differ considerably between studies published with and without pre-registration. Studies with pre-registration were conducted with much larger samples than studies without pre-registration, which might be due to the higher standards and sensitivity regarding statistical power, not only in recent years but also particularly with journals advocating pre-registration. By contrast, regarding within-subject designs, the sample sizes were smaller in studies with pre-registration than in studies without pre-registration. This makes the whole picture quite complicated because we would have expected the same influence of sensitivity regarding statistical power for both kinds of study design. One tentative explanation for this paradox might be that researchers, when conducting a replication study, indeed ran a power analysis that, however, yielded a smaller sample size than the original study had because within-subject studies generally have higher power.

**Table 2:** Median, mean, and SD of sample size, and percentage of significant effects for all studies where an effect size (*r*) was extracted or calculated.

All studies				Between-subjects designs				Within-subject designs			
*Mdn_N_*	*M_N_*	*SD_N_*	*% sig.*	*Mdn_N_*	*M_N_*	*SD_N_*	*% sig.*	*Mdn_N_*	*M_N_*	*SD_N_*	*% sig.*
**Studies with pre-registration**											
All disciplines											
268	1756	12424	64	358	2400	14730	63	71	181	295	65
											
**Studies without pre-registration**											
All disciplines											
89	364	1729	79	82	299	849	84	89	415	2198	74
Applied											
190	524	1892	84	120	214	253	100	190	616	2147	79
Biological											
32	132	715	78	36	221	1038	86	30	52	74	71
Clinical											
90	217	461	86	74	278	620	87	96	165	254	84
Developmental											
82	232	518	80	80	228	481	84	88	236	554	76
Educational											
103	453	1703	68	70	323	770	71	107	560	2192	66
Experimental											
42	86	221	73	70	106	137	83	30	77	252	68
Multidisciplinary											
160	400	793	83	154	428	955	88	160	370	589	71
Psychoanalysis											
91	387	1141	79	91	536	1510	77	89	219	412	83
Social											
150	847	4153	84	128	207	254	89	191	1680	6341	65

*Mdn_N_ = Median of sample size; M_N_ = Mean of sample size; SD_N_ = SD of sample size; % sig. = Percentage of significant effects (p < 0.05). Note that studies with pre-registration were too few to divide them into sub-disciplines.*

Table 2 also shows the percentage of significant effects, both for all studies and separately for studies with between-subjects and studies with within-subject designs (for studies published without pre-registration, in addition, for all sub-disciplines). The likelihood of obtaining a significant result was considerably smaller in studies published with pre-registration.

Fifty-one of the pre-registered studies (55%) were replication studies, six thereof multilab studies; the remaining 42 (45%) were original studies, one thereof a multilab study. Only 16 (17%) of the pre-registered studies explicitly stated that they were registered reports; all others just mentioned that there was a pre-registration at a specific repository.

### Empirical Distribution of Effects From Studies Without Pre-registration

**FIGURE 1:**
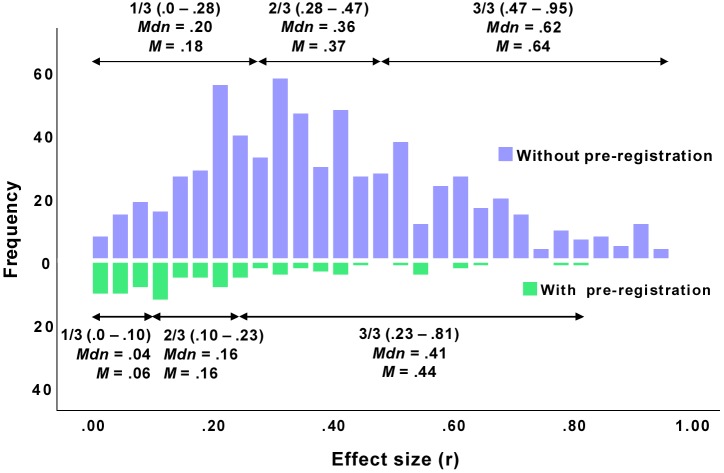
Distributions of effects (absolute values) from articles published with (*N* = 89) and without (*N* = 684) pre-registration. The distributions contain all effects that were extracted as or could be transformed into a correlation coefficient *r*.

Figure 1 (upper part) shows the empirical distribution of effects from psychological publications without pre-registration in general, and Table 3 provides the descriptive statistics. The distribution is fairly symmetrical and only slightly right-skewed, having its mean at 0.40 and its grand median at 0.36. That is, effects in psychology that have been published in studies without pre-registration in the past concentrate around a value of 0.36. This is fairly in accordance with Cohen who suggested a value of *r* = 0.3 for a medium effect. However, looking at the lower third of the distribution of *r* reveals that the lower median (i.e., the 16.65% quantile) is 0.20, twice as much as Cohen’s suggestion (*r* = 0.1). Similarly, the upper median (i.e., the 83.35% quantile) is 0.62, which is also larger than Cohen’s suggestion (*r* = 0.5).

**Table 3:** Descriptive statistics of empirical effects (all transformed to *r*) from studies published with and without pre-registration.

Descriptor	Studies without pre-registration			Studies with pre-registration		
	Total	Between-subjects designs	Within-subject designs	Total	Between-subjects designs	Within-subject designs
*N*	684	313	358	89	63	26
*M*	0.40	0.37	0.42	0.21	0.17	0.33
*SD*	0.21	0.20	0.22	0.19	0.15	0.22
Lower *Mdn* (≈ small effect)	0.20	0.18	0.21	0.04	0.04	0.09
Grand *Mdn* (≈ medium effect)	0.36	0.34	0.38	0.16	0.12	0.31
Upper *Mdn* (≈ large effect)	0.62	0.57	0.67	0.41	0.30	0.56

Effects from studies with a between-subjects design were significantly smaller than effects from studies with a within-subject design: *t*(669) = 3.21, *p* = 0.001 (see Table 3). Note that whereas the lower medians do not differ much (Δ*Mdn* = 0.03) the upper medians differ considerably (Δ*Mdn* = 0.1).

Not surprisingly, experimental (*Mdn_r_* = 0.37) and quasi-experimental (*Mdn_r_* = 0.40) studies revealed larger effects than correlational and other studies (*Mdn_r_* = 0.31). Clinical (*Mdn_r_* = 0.36) and non-clinical (*Mdn_r_* = 0.35) studies did not differ in the effects reported.

### Empirical Distribution of Effects From Studies With Pre-registration

Figure 1 (lower part) shows the empirical distribution of effects from psychological publications with pre-registration in general, and Table 3 provides the descriptive statistics. The distribution is considerably different from the distribution of the effects from studies without pre-registration in two respects. First, it is markedly right-skewed and suggests that the effects concentrate around a very small modal value. Second, the distribution is made up of markedly smaller values: It has its mean at 0.21 and its grand median at 0.16. That is, effects in psychology that have been published in studies with pre-registration concentrate around a value that is not only significantly smaller than the median of effects published without pre-registration, but also smaller than what Cohen had suggested as a medium effect (*r* = 0.3). Looking at the lower third of the distribution of *r* reveals that the lower median is 0.04, half the magnitude of Cohen’s suggestion (*r* = 0.1). Similarly, the upper median is 0.41, which is also smaller than Cohen’s suggestion (*r* = 0.5).

Effects from explicitly registered reports (*N* = 16, *M* = 0.18, *SD* = 0.21) were smaller than effects from pre-registered studies that did not explicitly state that they were registered reports (*N* = 73, *M* = 0.22, *SD* = 0.18), although this difference is not significant: *t*(87) = 0.96 (*p* = 0.34; note, however, the small sample size here).

Again, effects from studies with a between-subjects design were significantly smaller than effects from studies with a within-subject design: *t*(87) = 3.83, *p* = 0.003 (see Table 3). Note that whereas the lower medians do not differ much (Δ*Mdn* = 0.05) the upper medians differ considerably (Δ*Mdn* = 0.26). Interestingly, the medians of the within-subject design studies (0.09, 0.31, 0.56) are strikingly close to Cohen’s suggestions (0.10, 0.30, 0.50). Surprisingly, in contrast to the studies without pre-registration, experimental (*Mdn_r_* = 0.12) and quasi-experimental (*Mdn_r_* = 0.12) studies revealed smaller effects than correlational and other studies (*Mdn_r_* = 0.22).

**FIGURE 2:**
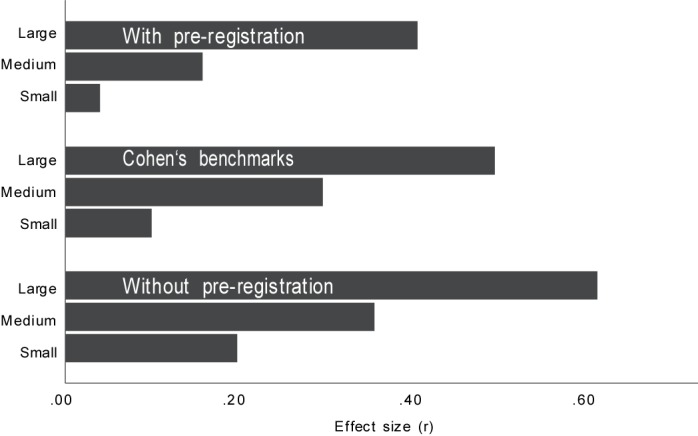
Cohen’s conventions for small, medium, and large effects compared with empirical effects (lower, grand, and upper median) from studies published with and without pre-registration.

In sum, the distributions of effects from published studies in psychology differ considerably between studies with and without pre-registration. While studies without pre-registration have revealed effects that were larger than what Cohen had previously suggested as a benchmark, studies with pre-registration, in contrast, revealed smaller effects (see Figure 2). In addition, it seems impossible to compare effects from studies with between-subjects designs and within-subject designs, particularly when it comes to large effects.

### Comparison of Psychological Sub-Disciplines

**FIGURE 3:**
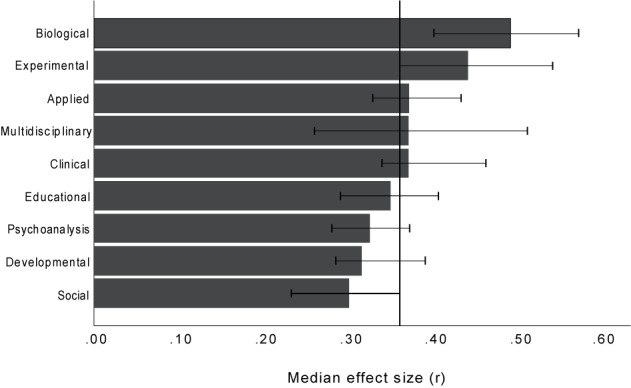
Medians (with 95% bootstrap confidence intervals) of effects published without pre-registration (absolute values) for the nine Social Sciences Citation Index psychological sub-disciplines. The bars contain all effects that were extracted as or could be transformed into a correlation coefficient *r*. The vertical line is the grand median.

For Cohen (1962, p. 146), it was essential that any general and fixed benchmarks should be treated with caution because researchers work in “diverse content areas, utilizing a large variety of dependent variables and many different types of statistical tests.” This concern is emphasized by a considerable variance in the effect size distributions of different psychological sub-disciplines. Figure 3 shows the medians and 95% confidence intervals of the effects sizes in the nine SSCI sub-disciplines. Note that this analysis could only be done for the studies published without pre-registration because studies with pre-registration were too few to be sensibly divided into sub-categories.

The largest effects come from disciplines such as experimental and biological psychology where the use of more reliable instruments and devices is common. Disciplines such as social and developmental psychology provide markedly smaller effects. Note that, for instance, there is not even an overlap of the confidence intervals of social and biological psychology. This simply means that in terms of effect sizes, we are talking about completely different universes when we talk about psychological research as a whole.

The differences between the sub-disciplines shown in Figure 3 largely match the differences between the results of the studies discussed in the Introduction. For instance, Morris and Fritz (2013) reported an unexpectedly large median effect of η^2^ = 0.18 for studies from memory research, which we categorized into experimental psychology. By contrast, Richard et al. (2003) reported an unusually small median effect of *r* = 0.18 for studies from social psychology.

### Influence of Potential Moderators on Effect Sizes

#### Sample Size

**FIGURE 4:**
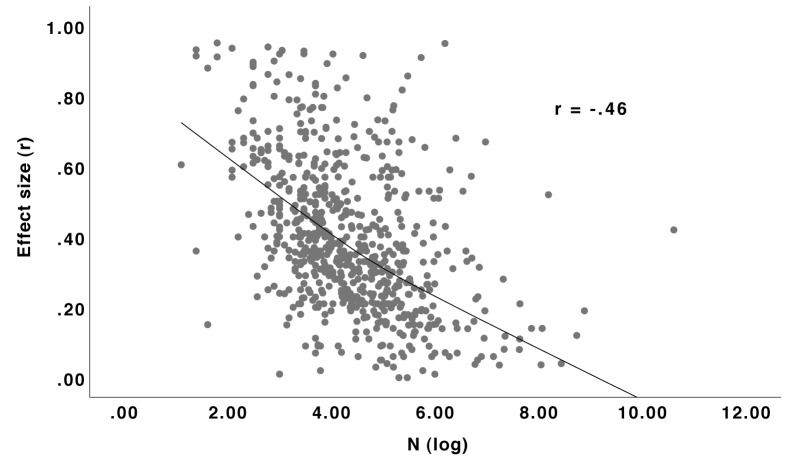
Relationship (Loess curve^1^) between sample size and effect size *r* for studies published without pre-registration. *N* = 684.

**FIGURE 5:**
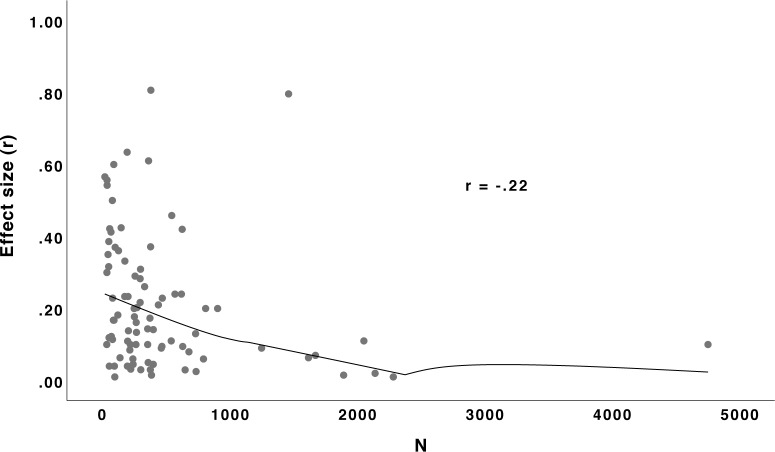
Relationship (Loess curve) between sample size and effect size *r* for studies published with pre-registration. *N* = 89.

Effect sizes were smaller the larger the samples (see Figure 4, 5). One obvious explanation for these strong correlations is the publication bias, since effects from large samples have enough statistical power to become significant regardless of their magnitude. However, a look at Figure 5 reveals that with studies published with pre-registration, hence potentially preventing publication bias, the correlation is indeed smaller but still far from zero. This result is in accordance with the result of the Reproducibility Project in Psychology (Open Science Collaboration, 2015), where for replication studies, the standard error was a significant predictor (*z* = 3.47, *p* < 0.001) for the observed effect size. The authors concluded that “[b]ecause publication bias was absent, this positive effect of standard error was likely caused by using power analysis for replication studies, i.e., generally larger replication samples were used for smaller true effects” (Open Science Collaboration, 2015, supplemental information). This general correlation between sample size and effect size due to statistical power might also have led to a learning effect: in research areas with larger effects, scientists may have learned that small samples are enough while in research areas with smaller effects, they know that larger samples are needed. Moreover, studies on social processes or individual differences can be done online with large samples; developmental studies can be done in schools, also providing large samples. By contrast, experimental studies or studies requiring physiological measurement devices are usually done with fewer participants but reveal larger effects. However, when calculating the correlation between sample size and effect size separately for the nine sub-disciplines, it is still very large in most cases (ranging from 0.05 for multidisciplinary to 0.62 for clinical). The relationship between larger effects and the use of more reliable measurement devices might of course also be there *within* the sub-disciplines but this explanation needs more empirical evidence.

#### Year of Publication

**FIGURE 6:**
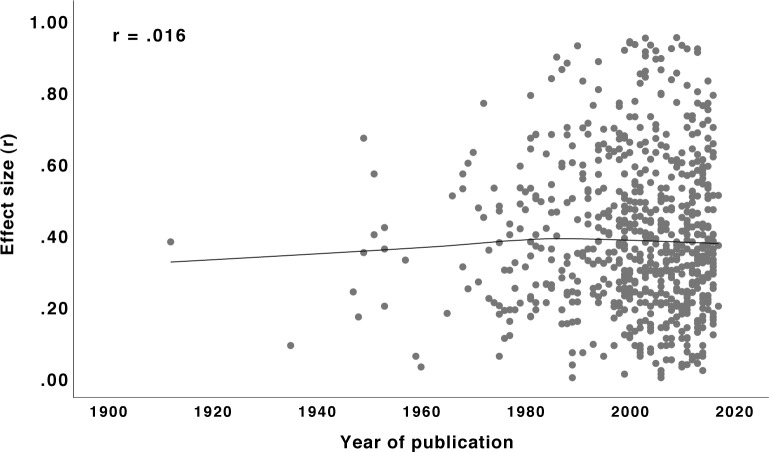
Relationship (Loess curve) between year of publication and effect size *r* for studies published without pre-registration. *N* = 684.

Is the year of publication associated with the effect size? For instance, the call for replication studies in recent years together with the decline effect (e.g., Lehrer, 2010) might have led to decreasing effect sizes. As Figure 6 shows, however, there is no correlation between year of publication and size of the effects reported (only done for studies published without pre-registration since studies with pre-registration started no earlier than 2014). Thus, effect sizes appear to be relatively stable over the decades so that, in principle, nothing speaks against providing fixed guidelines for their interpretation.

## Discussion

### The Impact of Analysis, Reporting, and Publication Biases on Published Effects in Psychology: Comparing Effects Is Not (Yet) Useful

When is an effect small or large? The present results demonstrate that this is not so easy to answer. As Cohen suggested in his seminal writings, the interpretation of an effect’s magnitude should be guided by the typical effects that have been found in the past in a specific area of research. Hence, it should make sense to look at the distribution of empirical effects that have been published in the past 100 years or so. We have called this the comparison approach. Yet, as shown, this does not seem to be a practicable solution because most published effects are seriously inflated because of the potential biases in analyzing, reporting, and publishing empirical results. The Open Science Collaboration (2015) found that the mean effect of 100 original studies diminished from *M_r_* = 0.40 to *M_r_* = 0.20 in direct high-powered replications. This is what we can verify by the present analysis: The median effect of studies published without pre-registration (i.e., potentially affected by those biases) of *Mdn_r_* = 0.36 stands in stark contrast to the median effect of studies published with pre-registration (i.e., very unlikely to be affected by the biases) of *Mdn_r_* = 0.16. Hence, if we consider the effect size estimates from replication studies or studies published with pre-registration to represent the true population effects we notice that, overall, the published effects are about twice as large. Notably, this contrast between effect sizes from studies with and without pre-registration primarily originates from studies with between-subjects designs: While the difference for these studies is Δ*Mdn_r_* = 0.34–0.12 = 0.22, it is only Δ*Mdn_r_* = 0.38–0.31 = 0.07 for studies using a within-subject design. One reason might be that within-subject designs generally have higher statistical power so that the effect of potential biases might be smaller.

The potential biases also seem to have affected the shape of the distribution of the effects: While the distribution of effects published without pre-registration is fairly symmetrical around its median, the distribution of effects published with pre-registration is markedly skewed and contains many more values close to zero. This is what one would expect given that in confirmatory (i.e., theory-driven) research a large number of hypotheses might be wrong (see Fanelli, 2012).

Thus, at least currently, the comparison approach is limited to the interpretation of an effect in the context of *published and potentially biased* effects only but it fails to provide a comparison with real population effects. In other words, one can compare the effect of a study with previous effects in the respective area of research but must keep in mind that these past publications provide a biased picture with effects much larger than what holds true for the population. The hope is, of course, that the near future will bring many more studies that adhere to a strict pre-registration procedure in order to prevent the potential biases (and other problems). Once there is a reliable basis of such studies in a couple of years, the comparison approach can develop its full potential—just as intended by Cohen years ago. For the time being, however, it might be wiser to interpret the size of an effect by looking at its unstandardized version and its real-world meaningfulness (see Cohen, 1988; Kirk, 1996; Baguley, 2009).

### Differences Between Psychological Sub-Disciplines: General Benchmarks Are Not Useful

An alternative way to interpret the size of an effect—besides comparing it with effects from the past—is to apply conventional benchmarks for small, medium, and large effects. Cohen had advocated the use of this conventions approach in cases where there are no or not many previous studies in a specific field of research; and many researchers use Cohen’s benchmarks because they are convenient and suggest a certain reliability. Cohen provided his now well-known conventions very hesitantly as he was aware that global benchmarks might not be applicable to all fields of behavioral sciences and there is the risk of overuse. Our analysis of the distributions of effects within psychological sub-disciplines revealed that Cohen was much more right than he may have thought: Effects differ considerably, partially in such a way that their confidence intervals do not even overlap. Whether publication bias has an influence on the size of these differences is unclear; more pre-registered studies are needed to reliably compare their effects between sub-disciplines. Nonetheless, these differences clearly speak against the use of general benchmarks. Instead, benchmarks should, if at all, be derived for homogeneous categories of psychological sub-disciplines. Again, the hope is that the future will bring many more pre-registered studies in all sub-disciplines to accomplish this task. Apart from that, we advocate the use of the comparison approach over the conventions approach whenever possible because the interpretation of an effect’s magnitude highly depends on the area of research and the specific research question. As Kelley and Preacher (2012, p. 146) conclude, “as tempting as it may be, the idea of linking universal descriptive terms (e.g., “small,” “moderate,” or “large”) to specific effect sizes is largely unnecessary and at times misleading.”

What also speaks against the use of general benchmarks is the difference between effects from within-subject versus between-subjects designs. Due to the omission of between-subjects variance, within-subject designs reveal considerably larger effects (see also the review of Rubio-Aparicio et al., 2018, for large differences between the two different designs).

### Limitations

#### Do Pre-registered Studies Provide a Picture of the Real Population Effects?

We cannot rule out that there might be a self-selection effect in researchers pre-registering their studies. Pre-registered studies are more common in more highly ranked journals, which might provide a biased selection of well-established and mostly experimental research paradigms (we indeed found that the share of experimental designs is much larger with pre-registered studies). This might cause published effects to even be the larger ones. In contrast, one might suspect that researchers pre-register a study when they expect their studied effects to be small, in order to ensure publication in any case. This might cause published effects to be the smaller ones. As said, we need more pre-registered studies in the future to say something definite about the representativeness of pre-registered studies.

With regard to the different kinds of pre-registration, we also found that there is a difference between studies that were explicitly registered reports and studies that were not. That is, we cannot rule out that published pre-registered studies that are not registered reports are still affected by publication bias at least to a certain degree.

#### Are the Nine SSCI Sub-Disciplines Representative of Psychological Research?

Any categorization of psychological sub-disciplines is vulnerable. We decided to use the SSCI since it is a very prominent index and provides a rather fine-grained categorization of sub-disciplines. In any case, we showed that differences in the effect sizes between the sub-disciplines are considerable.

#### Are the Extracted Effects Representative of Psychological Research?

As explained, from each study, we analyzed the first main effect that clearly referred to the key research question of an article. For articles reporting a series of several studies this procedure might cause a certain bias if the first effect reported happened to be particularly small or particularly large. To our knowledge, however, there is no evidence that this should be the case (although this might be a worthwhile research question on its own).

#### Are Analysis, Reporting, and Publication Biases the Only Cause of Differences Between Effects From Studies With and Without Pre-registration?

As we have argued throughout this article, biases in analyzing, reporting, and publishing empirical data (i.e., questionable research practices and publication bias) are most likely responsible for the differences between the effect sizes from studies with and without pre-registration. Yet, there is of course a remaining risk of other factors potentially being responsible for the difference between these two kinds of studies (over and above the factors discussed in section “Do Pre-registered Studies Provide a Picture of the Real Population Effects?”): the truth might “wear off” (see Lehrer, 2010) so that true effects might get smaller over the years; more recent research questions might have been particularly complicated or detailed so that they reveal smaller effects; there might be more hidden moderators in more recent and/or pre-registered research designs; researchers in psychology might have got worse or unlucky in recent years, or maybe only those who pre-register their studies—to list only some. Although we cannot rule out these potential factors and some of them might have a certain influence, we still believe that the lion’s share of the differences obtained in our study results from the potential biases discussed above. Having said this, we definitely recommend addressing the question of how pre-registered (or newer) studies might differ from conventional (or older) studies in future research.

## Conclusion

We can now draw conclusions regarding the two main focuses of effect sizes: answering research questions and calculating statistical power. We have shown that neither the comparison approach nor the conventions approach can be applied to interpret the meaningfulness of an effect without running into severe problems. Comparisons are hard to make when there is no reliable empirical basis of real population effects; and global conventions are useless when differences between sub-disciplines and between study designs are so dramatic. One pragmatic solution for the time being is something that Cohen (1988) himself had suggested: express effects in an unstandardized form and interpret their practical meaning in terms of psychological phenomena (see also Baguley, 2009)—thereby accepting the problem that unstandardized effects are hard to compare across different scales and instruments. We also expressed our hope for the future that many more pre-registered studies will be published, providing a more reliable picture of the effects in the population. We will then be able to really exploit the comparison approach. Moreover, separately for sub-disciplines and for between-subjects versus within-subject studies, new benchmarks could then be derived. As it stands now, it appears that Cohen’s benchmarks will have to be revised downward in general, at least for between-subjects studies’ effects.

Our finding that effects in psychological research are probably much smaller than it appears from past publications has an advantageous and a disadvantageous implication. When interpreting the effect of a single study, it is of course nice to know that many effects are rather small and hence one’s own effect does not stand out. On the downside, smaller effect sizes mean that the under-powering of studies in psychology is even more dramatic than recently discussed (e.g., Bakker et al., 2012; Fraley and Vazire, 2014) because smaller population effects would require even larger samples to produce statistical significance. Thus, our findings once more underline the necessity of power calculations in psychological research in order to produce reliable knowledge.

## Author Contributions

Both authors have contributed equally to the development of the study’s idea, data acquisition, data analysis, and writing of the manuscript.

## Conflict of Interest Statement

The authors declare that the research was conducted in the absence of any commercial or financial relationships that could be construed as a potential conflict of interest.

## Footnotes

1 Locally estimated scatterplot smoothing (Loess) was run with the smoothing parameter *f* = 0.99, meaning that 99% of neighboring data points were used for smoothing the regression curve.
